# West Nile virus in Ontario, Canada: A twelve-year analysis of human case prevalence, mosquito surveillance, and climate data

**DOI:** 10.1371/journal.pone.0183568

**Published:** 2017-08-22

**Authors:** Bryan V. Giordano, Sukhdeep Kaur, Fiona F. Hunter

**Affiliations:** 1 Centre for Biotechnology, Brock University, St. Catharines, Ontario, Canada; 2 Centre for Vector-Borne Disease, Brock University, St. Catharines, Ontario, Canada; 3 Entomogen Incorporated, St. Catharines, Ontario, Canada; 4 Department of Biological Sciences, Brock University, St. Catharines, Ontario, Canada; Centro de Pesquisas René Rachou, BRAZIL

## Abstract

West Nile Virus (WNV) first arrived in Ontario, Canada in 2001 and has since spread throughout most of the province, causing disease in humans. The provincial government established a province-wide surveillance program to monitor WNV transmission throughout the 36 regional health units. Here we have acquired records of WNV human and mosquito surveillance from 2002 to 2013 to describe seasonal and geographic trends in WNV activity in southern Ontario. Additionally, we obtained climate data from seven municipalities to investigate how temperature and precipitation affect WNV transmission dynamics. We identified a strong quadratic relationship between the number of confirmed human cases and positive *Culex* mosquito pools recorded at the end of each year (R^2^ = 0.9783, p < 0.001). Using Spearman rank correlation tests, we identified that the minimum infection rate of *Culex pipiens/restuans* pools are the strongest predictor of human cases at a 1 week lag period. We also identified positive correlations between minimum infection rates, temperature, vector abundance, and cumulative precipitation. Global Moran’s I index indicates strong positive autocorrelation and clustering of positive *Culex* pool counts in southern Ontario. Local indicators of spatial association tests revealed a total of 44 high-high and 1 high-low trap locations (n = 680). In the current work we have identified when and where hot spots of WNV activity have occurred in southern Ontario. The municipalities surrounding the western shore of the Lake Ontario and Windsor-Essex County have the largest records of positive mosquitoes and human cases. We identified that positive mosquitoes are a strong indicator of human cases to follow in the coming weeks. An epidemic action threshold of cumulative positive *Culex* pools was established, allowing Ontario public health officials to predict an epidemic at epidemiological week 34 (rho = 0.90, p < 0.001). These data have the potential to contribute to more efficient larvicide programs and awareness campaigns for the public.

## Introduction

Despite more than a decade of pesticide use and awareness campaigns, West Nile virus (WNV; Family Flaviviridae, genus *Flavivirus*), an arthropod-borne virus that is transmitted through the bite of an infected mosquito, continues to be the leading cause of mosquito-borne disease in Canada [1–3]. WNV is a member of the Japanese encephalitis virus serogroup along with other viruses that cause encephalitic disease in humans such as Japanese encephalitis virus and St. Louis encephalitis virus [4,5]. Humans occasionally become infected but are considered ‘dead-end’ hosts because they do not produce a high enough viremia to transmit the virus to uninfected mosquitoes [1]. If infection does occur in humans, the severity can vary greatly; ∼80% of infections are asymptomatic, ∼20% develop into West Nile fever, and < 1% develop into deadly neuroinvasive disease [6,7].

WNV was originally identified in 1937 from the blood of a woman living in the West Nile district of Uganda [8]. Following the introduction of WNV into New York City, USA in 1999 [9], the virus quickly spread through much of North and South America and was first detected in Ontario, Canada in September 2001. Since its arrival in Canada there have been over 5000 confirmed human cases, of which approximately one fifth are classified as WNV neurological disease [3,10]. The Public Health Agency of Canada (PHAC) estimates that an additional 18,000–27,000 human WNV cases may have occurred and gone unreported since most WNV cases are asymptomatic [3].

It is well established that WNV is involved in an enzootic cycle involving avian hosts and mosquitoes in the genus *Culex* [11–16]. Historically the clear majority of collected *Culex pipiens* Linnaeus and *Culex restuans* Theobald test positive for WNV, due to their selective preference for an avian blood meal [17–21]. These ornithophilic species are known to become attracted to humans primarily during the late summer months [16], thereby contributing to both the enzootic cycle in birds and tangential transmission in humans. Other genera with wide host ranges also test positive for WNV if they happen to feed upon an infected bird [12,15,22,23].

Numerous studies have shed light on factors that affect WNV transmission such as severity of the preceding winter, drought, rainfall, heatwaves, density of mosquito vectors, density of vertebrate hosts, landscape, and availability of mosquito breeding site abundance [15,24–27]. Most these studies were conducted in the USA where the WNV human case prevalence is much higher due to a variety of factors such as warmer summers, a larger number of mosquito vector species (compared to Canada), and presence of sub-tropical regions in the southern US, which is why researchers and health officials are still unable to adequately predict when and where epidemics will occur in Canada. Furthermore, a detailed study concerning the epidemiology of WNV in Canada, specifically that of southern Ontario, where the largest populations of Canadians reside [28], has not been attempted in nearly a decade. Ontario has since experienced another WNV epidemic in 2012, and nine mosquito species have been added to the list of endemic species [29,30], all important factors that should be considered in an assessment of WNV transmission dynamics in Ontario.

The goal of this paper was to utilize data from the Ontario mosquito surveillance program and available climate data to build a relevant model for Ontario public officials and HU staff to utilize as an early warning system for epidemics and to identify regions of WNV activity. In the current work we have compiled both mosquito surveillance and WNV human case prevalence from 2002 to 2013 from the Entomogen Inc. and the Public Health Ontario (PHO; the provincial governing body responsible for health protection and promotion) WNV databases. To explore WNV outbreaks in more detail we obtained data for weekly mosquito abundance, minimum infection rate (MIR), average temperature, average amount of precipitation, and cumulative average amount of precipitation for seven Ontario Public Health Units (HU; the municipal governing body responsible for administering health promotion and disease prevention programs): Durham region (DUR), Halton region (HAL), Niagara region (NIA), Peel regional (PEE), City of Toronto (TOR), Windsor-Essex County (WEC), and York region (YRK). This will be the first epidemiological analysis of Ontario WNV human case prevalence and mosquito surveillance data that includes multiple epidemic years.

## Materials and methods

### Study area

**Fig 1:**
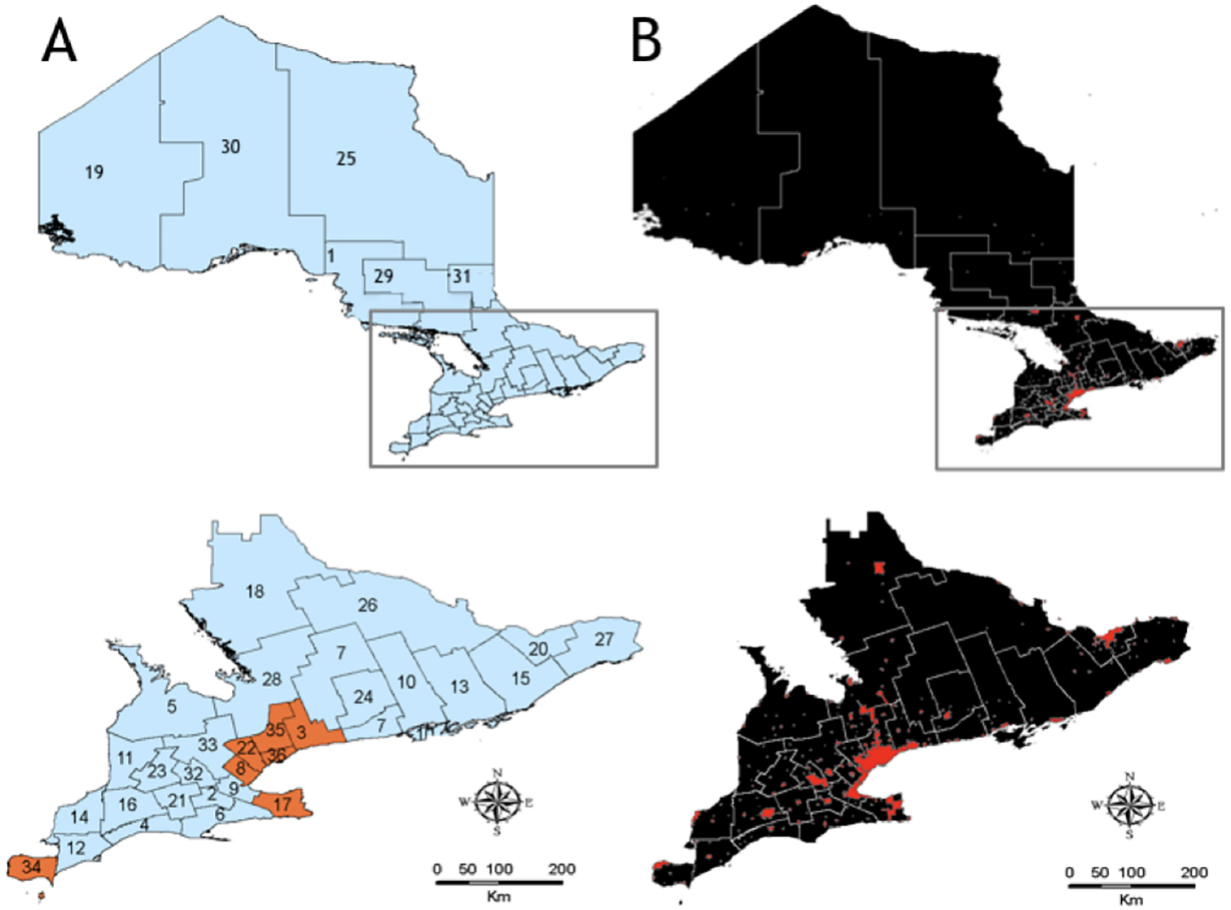
Map of the Ontario, Canada showing boundaries of municipal public health units and population density. (A) The 36 Ontario municipal public health units. Public health units we obtained weekly data from are highlighted in orange. 1-Algoma District; 2- Brant County; 3- DUR; 4- Elgin-St. Thomas; 5-Grey Bruce; 6- Haldimand-Norfolk; 7-Haliburton-Kawartha-Pine Ridge District; 8- HAL; 9-Hamilton; 10-Hastings and Prince Edward Counties; 11-Huron County; 12-Chatham-Kent; 13-Kingston-Frontenac and Lennox & Addington; 14-Lambton County; 15-Leeds-Grenville and Lanark District; 16-Middlesex-London; 17-NIA; 18-North Bay Parry Sound District; 19-Northwestern Region; 20-City of Ottawa; 21-Oxford County; 22-PEE; 23-Perth District; 24-Peterborough County-City; 25-Porcupine; 26-Renfrew County and District; 27- Eastern Ontario; 28 Simcoe Muskoka District; 29-Sudbury and District; 30-Thunder Bay District; 31-Timiskaming; 32-Waterloo Region; 33-Wellington-Dufferin-Guelph; 34-WEC; 35-YRK; 36- TOR. (B) Population centers are shown in red. Maps were created in Arc Map 10.4.

The province of Ontario is in the northern temporal zone. Ontario is Canada’s third largest province spanning 1.076 million km^2^ and most populous at a population of 13.6 million people. The largest human population densities in Ontario are localized to a few urban municipalities (City of Hamilton (HAM), City of Ottawa (OTT), TOR, DUR, HAL, NIA, PEE, WEC, and YRK) located in the southern region of the province (Fig 1). A population density boundary file was obtained from Statistics Canada Census 2011 database [31] and uploaded into ArcMap 10.4 (Esri). Population centers are defined as a minimum population concentration of 1000 persons and a population density of at least 400 persons per square kilometer [31].

### Mosquito and human surveillance data collection

In Ontario, the presence of WNV is monitored by the 36 HUs (Fig 1) and by the PHACs First Nations Inuit Health Branch [32,33]. Each year from May to October, Centres for Disease Control and Prevention (CDC) miniature light-traps baited with dry ice are set on a weekly basis consistent with the epidemiological weeks (epi-weeks) established by the CDC [34]. Light traps are collected the next day by HU staff and samples sent to a PHAC certified diagnostic laboratory for species identification and viral testing. Prior to testing, specimens collected from each light trap are sorted by species into pools comprising of no more than 50 individuals. *Aedes japonicus* (Theobald), *Aedes vexans* (Meigan), *Anopheles punctipennis* (Say), *Anopheles quadrimaculatus* (Say), *Anopheles walkeri* Theobald, *Cx*. *pipiens*, *Cx*. *restuans*, *Culex salinarius* Coquillett, *Culex tarsalis* (Coquillett), *Ochlerotatus canadensis* (Theobald), *Ochlerotatus stimulans* (Walker), *Ochlerotatus triseriatus* (Say), and *Ochlerotatus trivittatus* (Coquillett) are the mosquito species selected for testing for the presence of WNV by Real Time reverse transcriptase polymerase chain reaction.

Due to difficulties separating specimens of *Cx*. *pipiens* and *Cx*. *restuans* that have been damaged in the light traps, PHO guidelines state that these species are to be combined for viral testing. We refer to such species pools as *Culex pipiens/restuans* pools [32].

Human and mosquito surveillance records were obtained from Entomogen Inc. and the PHO WNV surveillance archive. Human cases are passively reported to the appropriate HU upon confirmation by plaque reduction neutralization assay by the PHAC. Both confirmed WNV neuroinvasive and non-neuroinvasive human cases were used in these studies. Travel related cases were omitted from the current work.

We obtained weekly mean mosquitoes per trap night for *Ae*. *vexans*, *Cx*. *pipiens/restuans*, *Cx*. *salinarius*, and *Och*. *stimulans* from 2002 to 2013. *Culex pipiens/restuans* and *Cx*. *salinarius* were selected since *Culex* species are known to be involved in the enzootic cycle of WNV, act as bridge vectors, and are abundant in the late summer, historically when most WNV human cases occur in Ontario [16,20,35,36]. *Aedes vexans* was selected as it is the second most common species pool to test positive for WNV, it is the most abundant of the thirteen WNV vectors, and populations in Ontario peak during the late summer months [17]. *Ochlerotatus stimulans* was selected to act as a negative control since this species population is known to be abundant in the early spring and populations diminish throughout the summer [17].

The weekly MIR for the DUR, HAL, NIA, PEE, TOR, WEC, and YRK HUs were calculated for positive *Cx*. *pipiens/restuans* pools from 2002 to 2013 as follows: MIR = [(Total No. positive pools) / (Total No. female mosquitoes tested)] * 1000 [37]. *Culex pipiens/restuans* pools were selected for the MIR calculation since they are the most common species pool to test positive for WNV in Ontario.

### Climate data collection

Observations of daily average temperature and daily total amount of precipitation were obtained from Environment Canada database of climate data [38]. Weekly averages of temperature and precipitation data were calculated in Microsoft Excel 2010. A total of thirty-one meteorological stations were selected. At least 3 meteorological stations were selected for each HU, except for PEE which only contained 2 stations with sufficient data. Weather stations selected for the current work are listed in S2 Table.

### Statistical analyses

Weekly and yearly totals of confirmed human cases and positive mosquito pools were tallied in Microsoft Excel 2010. Quadratic regression was performed on the total number of *Culex* pools and confirmed human cases recorded at the end of each year in Statistical Analysis Software (SAS; Statistical Analysis Software Institute Inc., NC, USA). Weekly data were further analyzed by Spearman rank correlation tests to measure the degree of linear correlation between confirmed human cases and average temperature, average amount of precipitation, mean number of mosquitoes per trap night, and the MIR; MIR and average temperature, average amount of precipitation, and mean number of mosquitoes per trap night; and mean number of mosquitoes per trap night and average temperature and average amount of precipitation. Lag periods of 0 to 6 weeks were tested to assess all relevant potential relationships. Lag periods larger than 7 weeks are not relevant; Spearman rank correlation tests did not produce significant results (p > 0.05) for lag periods larger than 7 weeks for this data set. An additional set of analyses was made using data from the WNV epidemic years (2002 and 2012). Spearman rank correlation coefficient (rho) ranges from -1 (strongly negatively correlated) to +1 (strongly positively correlated), and the null value of zero representing no correlation. Results were considered to be significant when p < 0.05.

In an effort to establish an action threshold for WNV epidemics we aligned the number of cumulative positive *Culex* pools and yearly totals of human cases each week, beginning with epi-week 24 (earliest recorded human case) and ending at 42 (end of surveillance program each year). These data were analyzed by Spearman rank correlation tests with lag periods ranging from zero to 12 weeks (end of August). We did not conduct analyses with lag periods larger than 12 as it is not practical for HUs and public health officials.

#### Geospatial analyses

The Global Positioning System (GPS) coordinates were obtained for each light trap that recorded a positive *Culex* mosquito pool. Only the HU label and date was obtained for each confirmed human case due to patient confidentiality. All GPS coordinates and HU labels were verified in ArcGIS^®^ software (Environmental Systems Research Institute Inc., Redlands, CA, USA). GPS coordinates were consolidated in Microsoft Excel 2010 to obtain a single coordinate associated with the sum of positive mosquito pools obtained from each light trap. These data were then uploaded into ArcMap 10.4 and plotted onto an Ontario HU boundary file [39] for geospatial analysis.

We used global Moran’s I index and local indicators of spatial association (LISA) to identify whether consolidated pool data are spatially autocorrelated due to bias in the sampling distribution, e.g., with more populous HUs having more extensive mosquito surveillance programs. Spatial autocorrelation tests were done using the Spatial Analyst Toolbox (ArcMap 10.4). We selected a zone of indifference weighting for our Moran’s index calculations. This method assigns a weight of 1.0 to any point within the specified search radius. The weight assigned to points located outside of the search radius decreases from 0.9 to 0.0 as the distance between the point and the search radius increases. These weights are assigned according to a Gaussian distribution. Global Moran’s index and p-value were recorded with 5, 10, 15, and 20 km search radii. Global Moran’s index ranges from -1 (data are dispersed) to +1 (data are clustered) [40]. The search radii with the largest positive global Moran’s index was selected as the bandwidth to study spatial clusters. LISA analyses assign traps a local Moran’s index and a p-value. Significance is considered at p < 0.10 for local Moran’s index [41]. Non-significant (NS; p > 0.10) point locations were assigned. All significant point locations (p < 0.10) were further classified by local Moran’s index and value of surrounding neighbours. When the local Moran’s index is greater than zero this indicates both high-high (HH) clusters, high values that occur near surrounding high values, and low-low (LL) clusters, low values that occur near surrounding low values [41]. If local Moran’s index is less than zero this indicates spatial outliers including high-low (HL), high values occurring near surrounding low values, and low-high (LH), low values occurring near surrounding high values [41].

If positive spatial autocorrelation was observed at small search radii (5 and 10 km) we proceeded with exploratory spatial data analysis to identify the distribution of the data set, describe spatial autocorrelation in more detail, and ensure the most appropriate geostatistical analysis was selected for interpolation. Spatial data analyses were performed in ArcMap 10.4 with the Geostatistical Analyst extension. We selected Empirical Bayesian Kriging, as it involves a distribution of semivariograms instead of a single model (accounting for error introduced during each semivariogram estimate), is known to produce more accurate predictions for normal or Gaussian distributed data sets and data sets that cover large areas, and produced optimal prediction errors (root mean squared standardized approximately equal to one, mean standardized approximately equal to zero, and root mean square nearest to the average standard error and less than 20) [42,43]. Interpolated maps of predicted mean number of positive pools and the associated standard error were overlaid to the Ontario HU boundary file.

The population for each southern Ontario HU was obtained from the Statistics Canada Census Database [44]. Confirmed human cases are presented as prevalence per 100,000 persons.

### Data availability

Data obtained from the Ontario province-wide mosquito surveillance program are available by request from PHO (http://www.publichealthontario.ca/en/About/Pages/Data.aspx). Trap locations and GPS coordinates of confirmed human cases cannot be disclosed as per the Ontario Personal Health Information Protection Act.

## Results

West Nile virus was first detected in Ontario, Canada in PEE on 31 August 2001 from a *Cx*. *pipiens/restuans* pool. From 2002 to 2013 the province of Ontario reported 2175 WNV positive pools including 1,892 (87.0%) *Cx*. *pipiens/restuans*, 134 (6.2%) *Ae*. *vexans*, 28 (1.3%) *Coquillettidia perturbans* (Walker), 28 (1.3%) *Cx*. *salinarius*, 23 (1.1%) *An*. *punctipennis*, 21 (1.0%) *Och*. *triseriatus*, 20 (0.9%) *Och*. *trivittatus*, 18 (0.8%) *Ae*. *japonicus*, 4 (0.2%) *An*. *quadrimaculatus*, 2 (0.1%) *Och*. *excrucians* (Walker), 2 (0.1%) *Och*. *stimulans*, 1 (< 0.1%) *An*. *walkeri*, 1 (< 0.1%) *Culiseta melanura* (Coquillett), and 1 (< 0.1%) *Ochlerotatus sollicitans* (Walker) (Table 1). An additional 189 positive pools were recorded at the level of genus and were omitted from the current work. We selected *Culex* pools for further analyses since this genus made up 88.3% of all positive pools.

**Table 1:** Number of WNV positive pools by species recorded in Ontario, Canada from 2002 to 2013.

Species	‘02	‘03	‘04	‘05	‘06	‘07	‘08	‘09	‘10	‘11	‘12	‘13
***Ae*. *japonicus***	0	0	0	0	0	1	1	1	3	9	2	1
***Ae*. *vexans***^***a***^	41	3	3	17	17	10	3	2	5	15	10	8
***An*. *punctipennis***	11	1	0	1	0	0	0	0	0	6	3	1
***An*. *quadrimaculatus***^***b***^	0	0	0	0	0	0	0	0	0	4	0	0
***An*. *walkeri***	0	1	0	0	0	0	0	0	0	0	0	0
***Cq*. *perturbans***	18	1	2	2	4	0	0	0	0	0	0	1
***Cx*. *pipiens/restuans***	301	105	60	260	156	38	56	11	48	237	436	184
***Cx*. *salinarius***	23	1	0	0	0	0	0	0	0	0	4	0
***Cs*. *melanura***	0	0	0	0	0	0	0	0	1	0	0	0
***Och*. *excrucians***	2	0	0	0	0	0	0	0	0	0	0	0
***Och*. *sollicitans***	0	0	0	1	0	0	0	0	0	0	0	0
***Och*. *stimulans***	0	1	0	0	0	0	0	0	0	1	0	0
***Och*. *triseriatus***	8	1	0	0	0	1	2	0	0	8	1	0
***Och*. *trivittatus***	11	1	1	2	3	0	0	0	0	1	1	0
**Total**	415	115	66	283	180	50	62	14	57	281	457	195

^a^ May include some specimens of *Aedes vexans nipponi* [45]

^b^ Specimens of the *An*. *quadrimaculatus* species complex were identified morphologically to *An*. *quadrimaculatus sensu lato*

**Fig 2:**
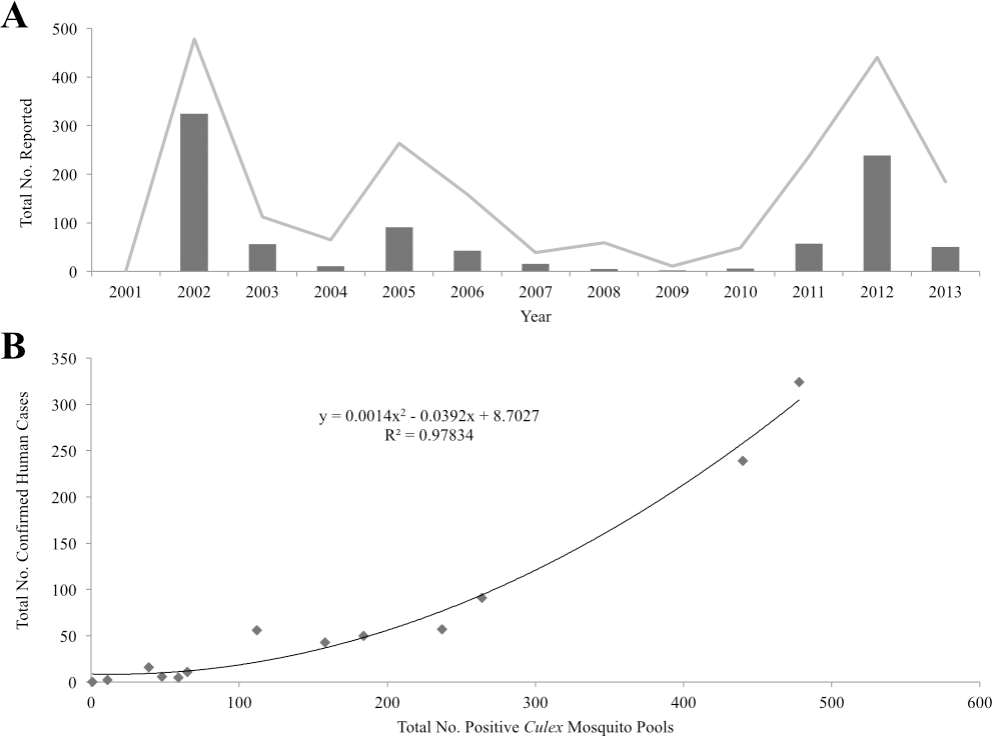
WNV human case prevalence and number of recorded *Culex* mosquito pools in Ontario, Canada from 2002 to 2013. (A) Comparison of confirmed WNV human cases and positive *Culex* mosquito pools recorded each year. Solid line represents WNV positive *Culex* mosquito pools. Bars represent confirmed WNV human cases. (B) Same data as in (A). A strong (R^2^ = 0.9783, p < 0.001) quadratic relationship between the total number of human cases and positive *Culex* pools recorded at the end of each field season in Ontario, Canada was observed.

During this study a total of 900 confirmed WNV human cases were reported by the PHAC. Fig 2A illustrates the number of recorded WNV confirmed human cases and positive *Culex* mosquito pools each year. Peak collections of both confirmed human cases and positive *Culex* pools were observed in 2002 (324 and 478) and 2012 (239 and 440) respectively. A strong quadratic relationship was identified between the number of confirmed human cases and positive *Culex* mosquito pools recorded at the end of each year (R^2^ = 0.9783, p < 0.001; Fig 2B). The total number of confirmed human cases, positive *Culex* mosquito pools, and positive non-*Culex* mosquito pools recorded in each HU are presented in S1 Table.

**Fig 3:**
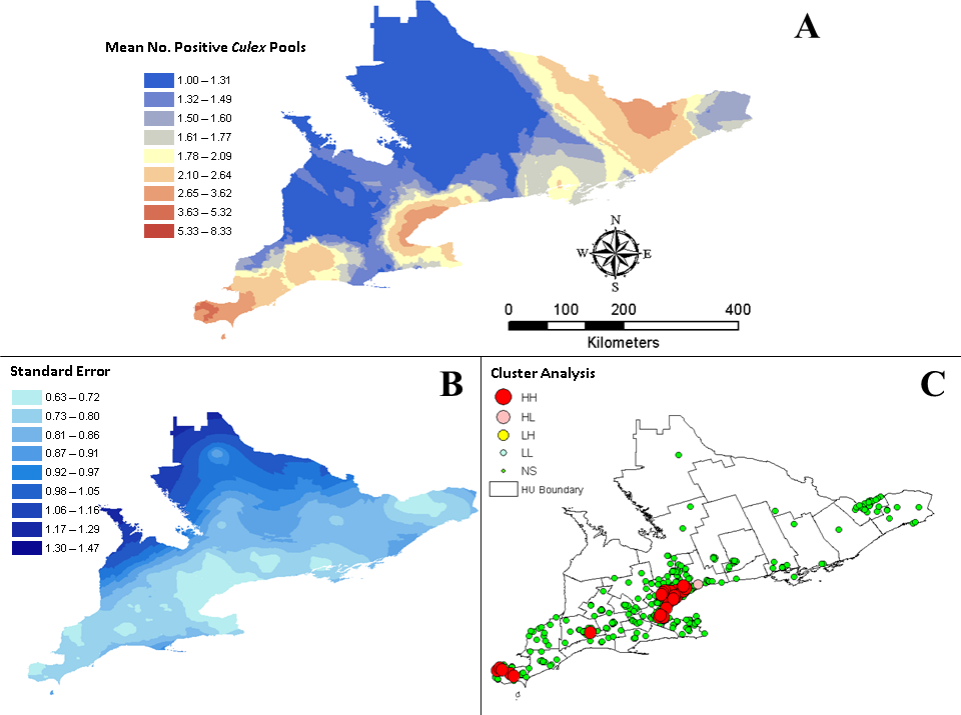
Geospatial analysis of WNV positive *Culex* mosquito pools in Ontario, Canada from 2002 to 2013. (A) Predicted mean number of positive *Culex* mosquito pools. (B) Standard error of predicted mean number of positive *Culex* pools. (C) LISA cluster analysis of WNV positive light traps recorded in Ontario during 2002 to 2013. LISA cluster analysis revealed both significant HH (local Moran’s index > 0, p < 0.05) and HL (local Moran’s index < 0, p < 0.05) trap locations (p < 0.05) as well as non-significant (NS, p > 0.05) trap locations (n = 680).

Exploratory spatial data analysis revealed that the data set is normally distributed when log-transformed. All recorded prediction error parameters are within acceptable ranges for accurate prediction interpolation (root mean square standardized = 1.054; mean standardized = 0.048; root mean square = 1.360; average standard error = 1.273). Predicted mean number of positive *Culex* pools and the calculated standard error are presented in Fig 3A and Fig 3B. Predicted mean number of *Culex* pools was the largest in DUR, HAL, HAM, PEE, TOR, WEC, and YRK (Fig 3A). Global Moran’s index for the 5 km (0.20, p < 0.001), 10 km (0.47, p < 0.001), 15 km (0.43, p < 0.001), and 20 km (0.38, p < 0.001) threshold distances all indicate strong positive autocorrelation and clustering of positive *Culex* pool counts in southern Ontario. For the LISA cluster analysis, we selected the threshold distance with the largest Moran’s index. Cluster analysis of trap locations with positive *Culex* pools are shown in Fig 3C. LISA analysis identified a total of forty-four HH and one HL trap location (n = 680) all located within the DUR, HAL, MSL, PEE, TOR, WEC, and YRK HUs (Fig 3C).

**Fig 4:**
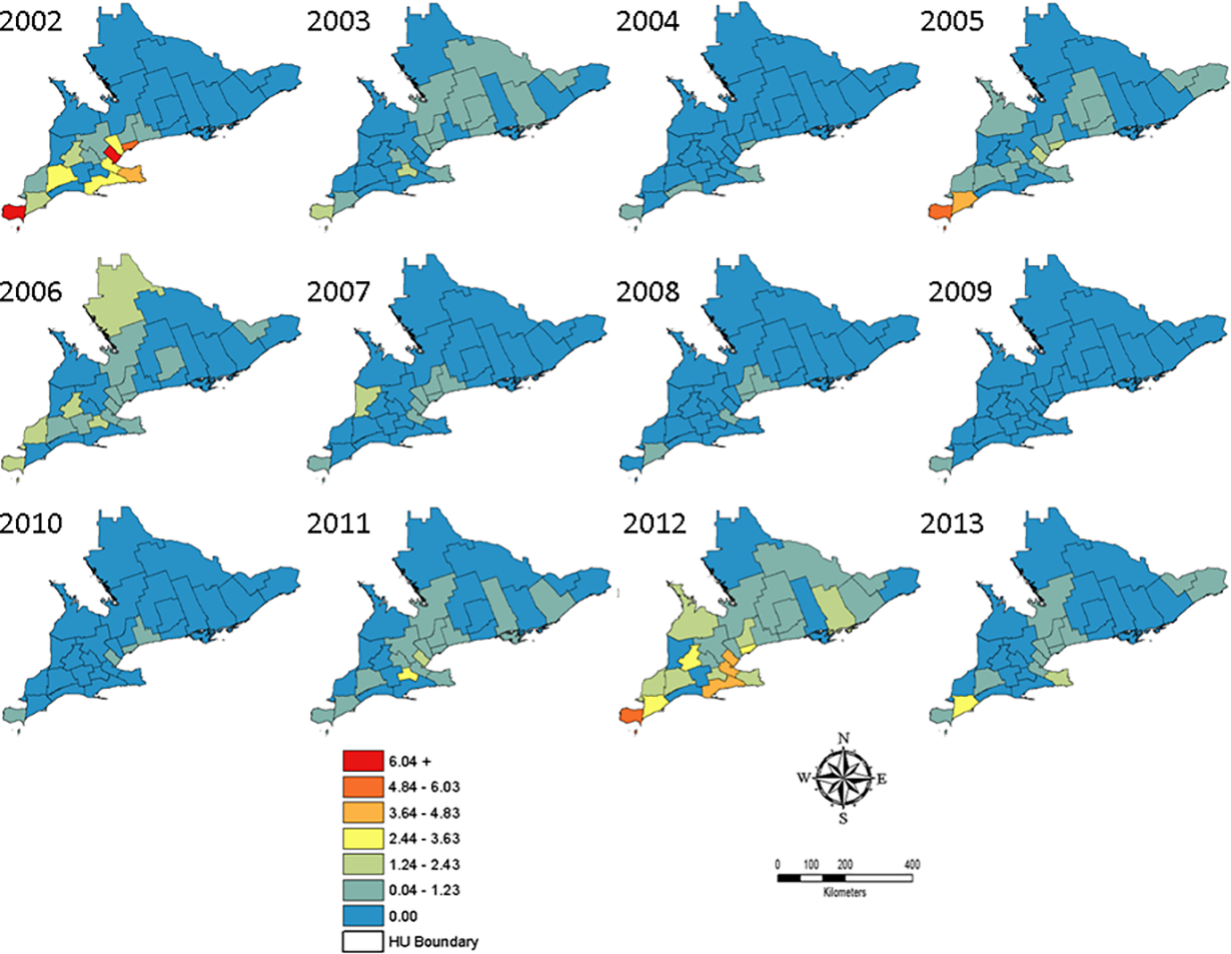
WNV human case prevalence per 100,000 persons in southern Ontario, Canada from 2002 to 2013. Point locations were not provided to the authors to protect the privacy of those who became infected. Maps were created in ArcMap 10.4.

Distribution maps for confirmed WNV human cases are presented in Fig 4 as prevalence per 100,000 persons. Human cases were recorded as far north as REN in 2003 and 2012 (Fig 4). No locally acquired confirmed human cases have been recorded in the northern Ontario HUs to date. The majority of WNV confirmed human cases typically occur in HAL, HAM, PEE, TOR, WEC, and YRK, with the vast majority of cases occurring in TOR (Fig 4). The largest recorded prevalence occurred in 2002 from HAL (15.46 per 100,000 persons) and WEC (9.33 per 100,000 persons).

**Fig 5:**
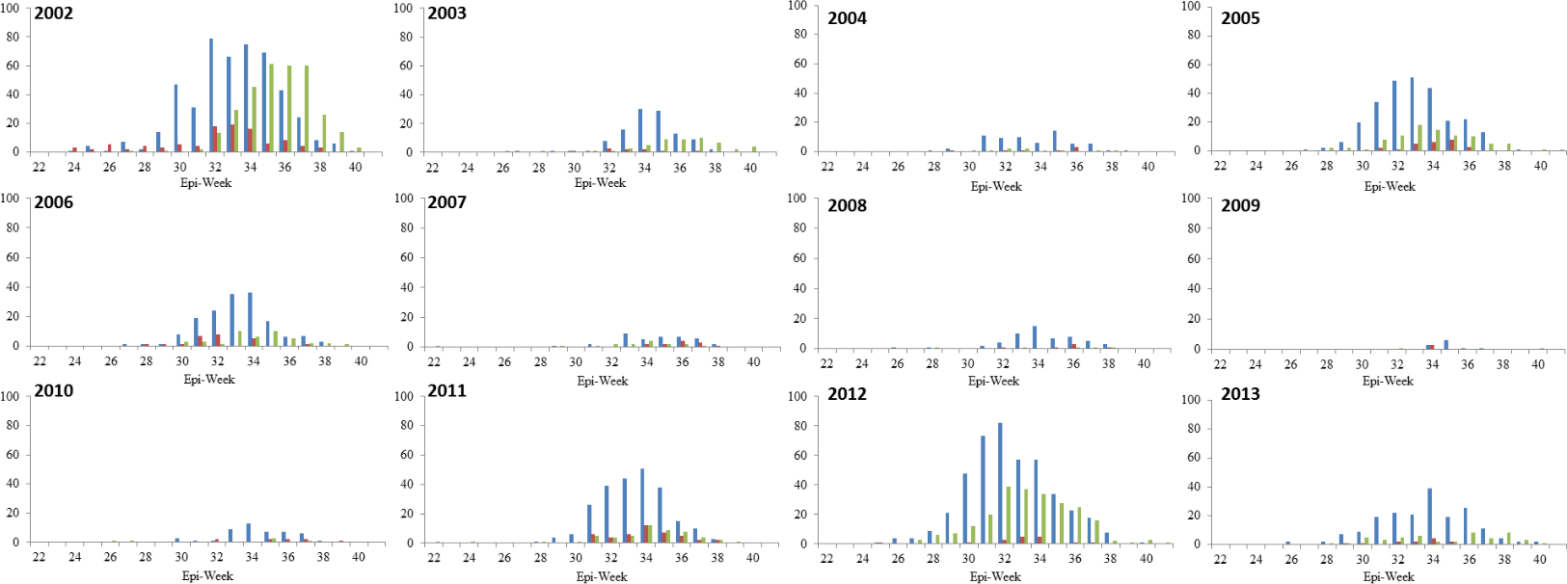
Epidemiological graphs of WNV surveillance data in southern Ontario, Canada from 2002 to 2013. Blue–WNV positive *Culex* mosquito pools; green–confirmed human cases; red–WNV positive non-*Culex* mosquito pools.

Seasonal distribution of confirmed WNV human cases and positive mosquito pools are presented in Fig 5. Mosquitoes from other genera (non-*Culex* pools) test positive each year, with a similar distribution as the *Culex* pools, however, in much lower numbers (Fig 5). Human cases typically begin to occur in late August and into September, corresponding to epi-weeks 32 to 36 (Fig 5). Upon initial observation, we identified an approximately 1 to 3 week lag period between the peak number of *Culex* pools and peak number of confirmed human cases (Fig 5). This pattern was observed during 2002, 2003, 2005, 2011, 2012, and 2013 (Fig 5).

**Fig 6:**
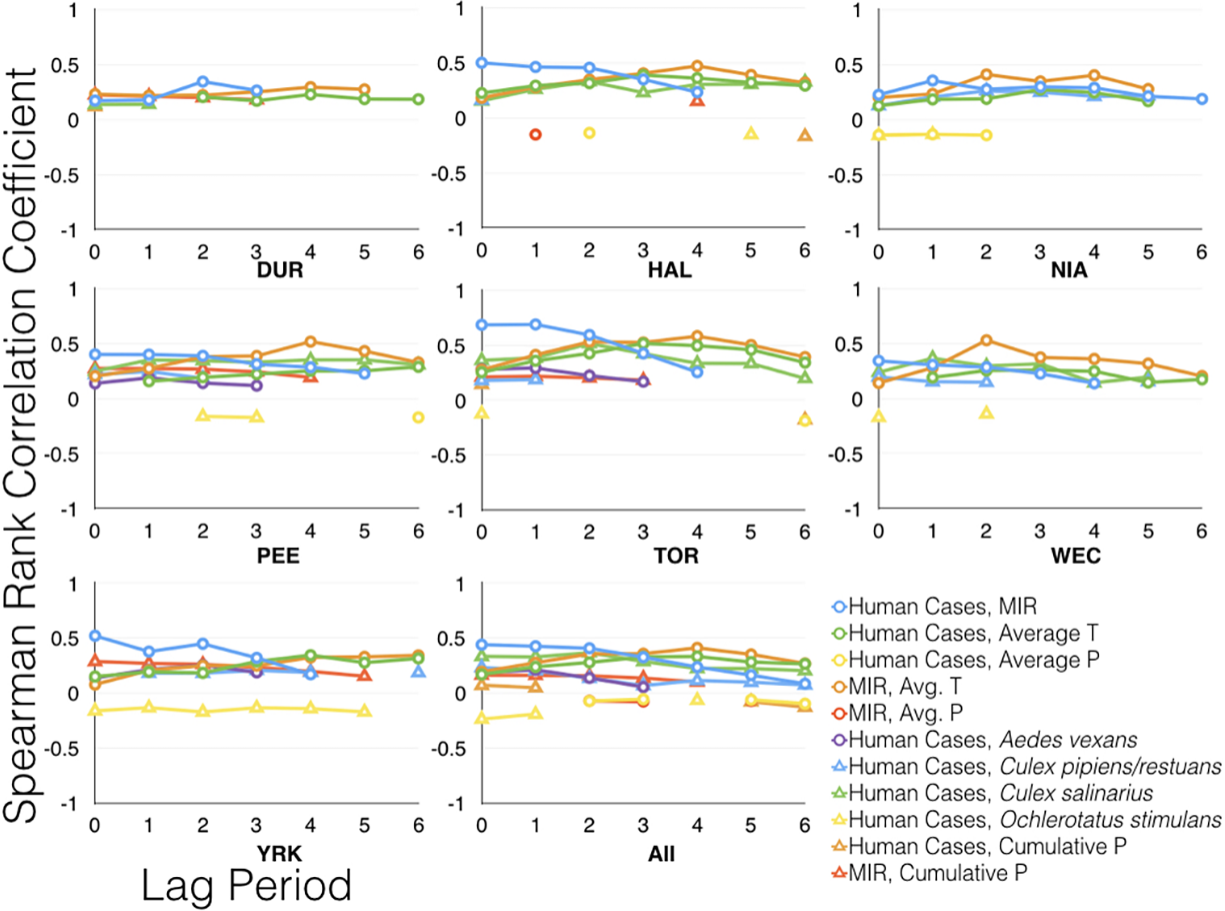
Spearman rank correlation coefficients for analyses including all years, 2002 to 2013. All, combined data from all HUs. Only significant (p < 0.05) data are presented.

**Fig 7:**
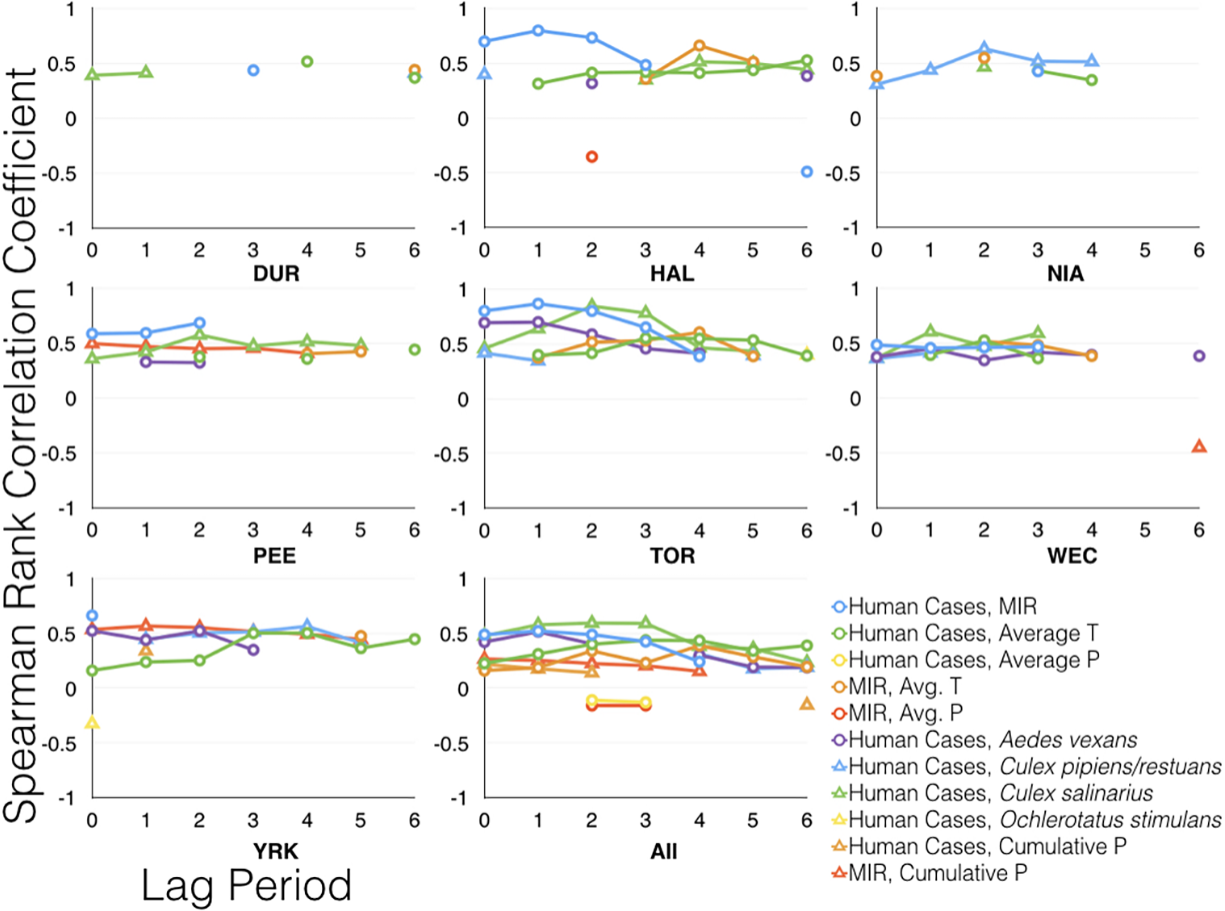
Spearman rank correlation coefficients for analyses of WNV epidemic years, 2002 and 2012. All, combined data from all HUs. Only significant (p < 0.05) data are presented.

Spearman rank correlation test results are presented in S3 Table. Significant results (p < 0.05) are summarized in Fig 6 and Fig 7. MIR was the strongest predictor of confirmed human cases in both all year (2002–2013) and epidemic year (2002 and 2012) analyses for HAL, PEE, TOR, and YRK. TOR exhibited the strongest correlation in all years (rho = 0.68, p < 0.001) and also in the epidemic years (rho = 0.87, p < 0.001) analyses with a 1 week lag period. The other four HUs exhibited weak to moderate positive correlation (0.34 < rho < 0.48, p < 0.05). During the epidemic years mosquito abundance was strongly correlated to human cases in the following HUs: NIA (*Cx*. *pipiens/restuans*; rho = 0.63, p < 0.001), TOR (*Cx*. *salinarius*; rho = 0.85, p < 0.001), WEC (*Cx*. *salinarius*; rho = 0.60, p < 0.001, YRK (*Cx*. *pipiens/restuans;* rho = 0.56, p < 0.001), and all HUs (*Cx*. *salinarius*; rho = 0.59, p < 0.001) (Fig 7).

Weekly average precipitation was not a good predictor of human cases at any lag period. Weekly cumulative average precipitation was only able to moderately predict both human cases and MIR during the epidemic years. TOR and YRK exhibited weak positive correlation between cumulative average amount of precipitation and human cases at lag 0 (rho = 0.35, p < 0.05) and lag 1 (rho = 0.34, p < 0.05), respectively. MIR and cumulative average amount of precipitation typically exhibited low to moderate positive correlation (0.15 < rho < 0.29, p < 0.05) when all years were considered. Moderate positive correlation was observed in PEE (rho = 0.50, p < 0.05) and YRK (rho = 0.57, p < 0.001) during the epidemic years at the 0 and 1 week lag period respectively.

Average temperature was a stronger predictor of MIR than average amount of precipitation in both sets of analyses (Figs 6 and 7). The strongest correlations were observed at a four to six week lag for DUR, HAL, PEE, TOR, and YRK and at two-week lag for NIA and WEC, and indicate weak to strong positive correlation (0.27 < rho < 0.66, p < 0.05) (S3 Table). Average amount of precipitation yielded insignificant results (p > 0.05) for NIA, PEE, TOR, WEC, and YRK, indicating that weekly precipitation data are not monotonic. A weak negative correlation was observed in HAL (rho = -0.36, p < 0.05) during the epidemic years with a two-week lag. A moderate positive correlation was observed in DUR at lag 6 (rho = 0.44, p < 0.05) and YRK at lag 5 (rho = 0.47, p < 0.05).

**Fig 8:**
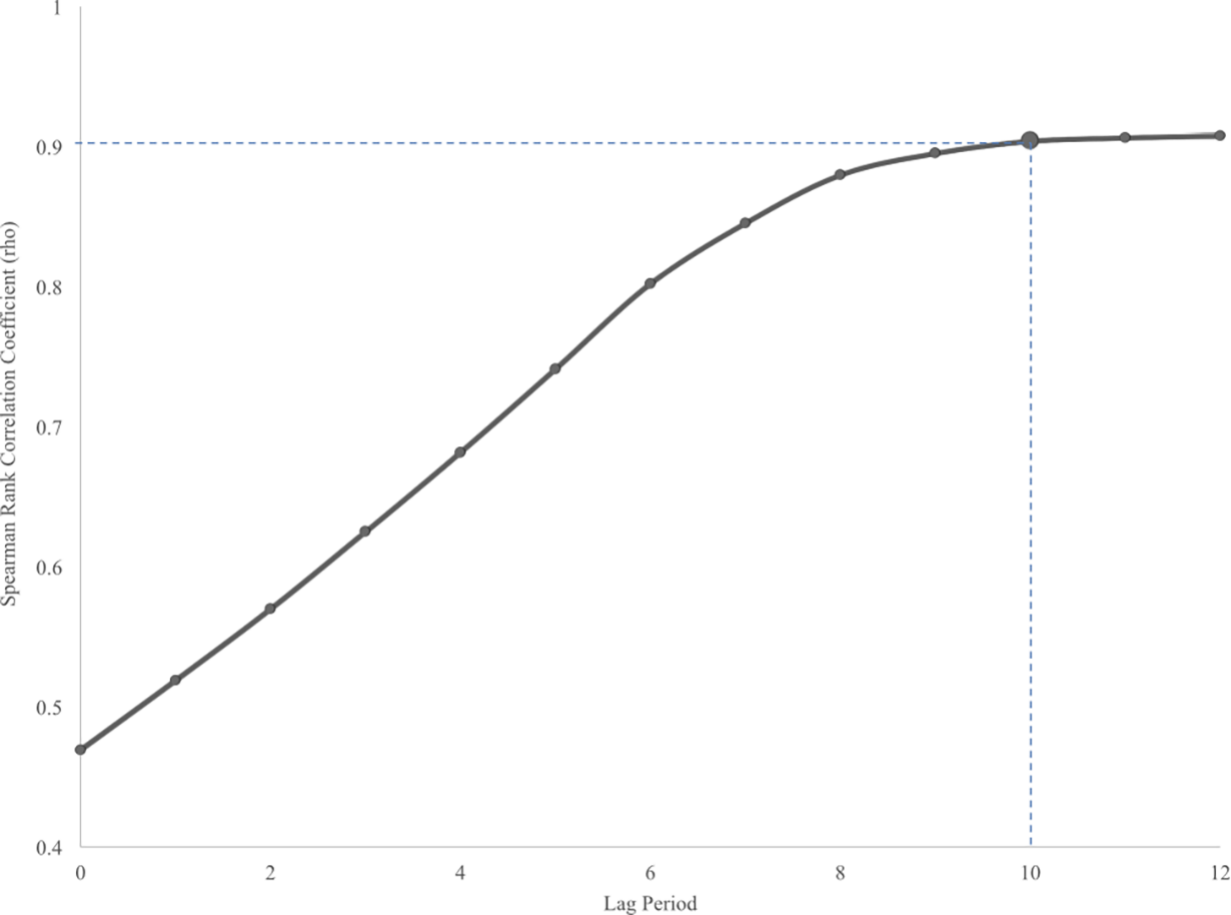
Predictive ability of cumulative positive *Culex* pools for confirmed WNV human cases in Ontario, Canada. All data were significant (p < 0.001)

The predictive ability of cumulative *Culex* pool counts is displayed in Fig 8. We identified that the Spearman Rank correlation coefficient given a ten-week lag in data was 0.90 (very strongly correlated) and only slightly increased to 0.91 by lag twelve, indicating that the cumulative number of *Culex* pools recorded by epi-week 34 is a sufficient action threshold for WNV epidemics is Ontario. Epi-week 34 corresponds to the second last week of August.

## Discussion

West Nile virus epidemics in Canada are difficult to study due to relatively low human case prevalence, large variations in the severity of outbreaks from year to year, and temperature dependency. Temperature is known to affect the extrinsic incubation period, mosquito metabolism, and mosquito survival. Hourly, daily, and weekly fluctuations in temperature and precipitation make it difficult to accurately assign these data to the epi-week calendar. This will continue to be a challenge for researchers and health officials alike since the province-wide mosquito surveillance program operates in accordance with the epi-week calendar. An additional challenge in monitoring WNV epidemics is passive human surveillance. Eighty percent of WNV infections are asymptomatic and WNV fever does not require immediate medical attention leading to potential delays in confirming presence of virus and a vast underestimation of cases. Onset of symptoms can develop between 2 and 12 days, which is consistent with our findings that demonstrate the strongest linear correlations between human cases and MIR range from 0 to 2 weeks (Figs 6 and 7). Other factors known to contribute to the underestimation of WNV human case prevalence such as socio-economic status, access to health care, and education are beyond the scope of this study.

Early studies in Ontario following the 2002 epidemic suggest the principal vectors to be mosquitoes of genus *Culex*, specifically ornithophilic *Cx*. *pipiens* and *Cx*. *restuans*. Kilpatrick et al. [16] suggests that *Cx*. *pipiens* act as both enzootic vectors, amplifying infection among the local bird populations, and bridge vectors, spreading infection to dead end hosts such as humans. Epidemics occur when adequate amplification in the bird population occurs early in the summer months with sustained above average daily temperatures, increasing the likelihood a mosquito vector with a wide host feeding preference would happen to feed on an infected bird, survive the extrinsic incubation period, and then seek out and feed upon a human host. Additionally, Kilpatrick et al. [16] and Russell and Hunter [35] observed that *Cx*. *pipiens* shifts its feeding preference from birds to humans in the late summer months. By monitoring increases in MIR and cumulative number of *Culex* pools early in the season we can infer that viral amplification is also occurring in the avian populations and determine whether spill over to humans is likely.

We have identified a very strong relationship between the number of human cases and the MIR in TOR, where the largest number of confirmed cases are recorded historically. Our analyses also identified a strong correlation between human cases and *Cx*. *salinarius* abundance during the two epidemic years; 2002 and 2012 recorded the largest numbers of positive *Cx*. *salinarius* pools (Table 1), suggesting that this species contributed to Ontario’s two epidemics. Hunter et al. [30] also noted peak collections of *Culex erraticus* (Dyar and Knab) in 2012, a vector for WNV that has expanded its North American range into Ontario in the early 2000s. *Culex erraticus* is known to feed on a wide variety of hosts and is an efficient laboratory vector for WNV. However, this species is not yet part of the province-wide surveillance program so its involvement in WNV transmission in Ontario remains to be elucidated.

We identified a moderate to strong correlation between temperature and MIR with a four to five-week lag period. This is consistent with established timelines of larval development, adult feeding preparation, ovarian development, and viral incubation period. Larval development typically requires one to two weeks and newly emerged adults require approximately four days to prepare for their first blood meal. After a successful bloodmeal from a WNV-infected avian host, the virus requires an extrinsic incubation period (dependant on temperature and host species) to replicate and disseminate throughout the mosquito host. Increased temperatures can reduce the extrinsic incubation period and larval and ovarian development time, fuelling downstream increases in mosquito abundance and subsequent increases in infection rates.

In the current study, we have identified when and where hot spots of WNV activity occur in southern Ontario. Our prediction surface is consistent with Beroll et al. [46] who also identified the greater Toronto area (DUR, HAL, HAM, PEE, TOR, and YRK) and WEC as hot spots of WNV activity. Our kriging estimates compliment the LISA cluster analysis, reaffirming that each year WEC and the greater Toronto HUs are hot spots for WNV positive mosquito vectors. We selected kriging as the method for interpolation as it considers spatial autocorrelation and produces a standard error surface. The standard error interpolation surface provides a visual check of the accuracy of the prediction model. Our prediction surface can be used to estimate the number of positive *Culex* pools any given trap would record at the end of each season. These data are consistent with our Choropleth maps of human case prevalence.

Here we report that the cumulative number of *Culex* positive pools at epi-week 34 can be used as an action threshold for WNV epidemics in Ontario. Our data suggest a very strong correlation (R^2^ = 0.9783, p < 0.001) to the total number of human cases reported at the end of each field season. Each year the estimated total number of confirmed human cases can be extrapolated from the quadratic regression equation we present.

Surveillance programs enable researchers and health officials to monitor species abundance, arboviral seasonal and spatial distributions, and the spread of invasive species. Since 2005, nine species have been added to the endemic mosquito checklist of Ontario, including *Cx*. *erraticus* a known vector for WNV [29]. Without a well-established mosquito surveillance program vectors of arboviral disease would go unnoticed until an outbreak or epidemic occurs. These programs also allow for estimations of species’ infection rates and the determination of high and low risk regions. Knowledge of these variables are of the utmost importance to determine the role any species plays in the endemic transmission of WNV [12]. Additionally, these data have the potential to contribute to a more efficient larvicide program (that targets specific species in high risk regions) and timely awareness campaigns. Given that taking protective measures to reduce exposure to mosquito bites can decrease the risk of contracting mosquito-borne disease [47,48], informing the public in a timely manner should continue to be the focus of mosquito surveillance programs.

## Supporting information

S1 Table Number of recorded confirmed human cases and positive mosquito pools in each Ontario HU from 2001 to 2013. CHC, confirmed human cases; PCMP, positive *Culex* mosquito pools; PNCMP, positive non-*Culex* mosquito pools.
(XLSX)

S2 Table Localities of the Environment Canada weather stations used for the collection of daily temperature and precipitation data in the current work. ^a^ Due to limited Environment Canada metrological weather stations with sufficient data located in PEE only 2 weather stations were selected.
(XLSX)

S3 Table Spearman rank correlation test results. ALL, all collected data from 2002 to 2013; MIR, minimum infection rate; ‘02‘12, data from the epidemic years 2002 and 2012 only. Boldface identifies the strongest correlation at the 95% confidence level. ^a^ denotes p < 0.05. ^b^ denotes p < 0.001.
(XLSX)
